# Drug-resistant tuberculosis treatments, the case for a phase III platform trial

**DOI:** 10.2471/BLT.23.290948

**Published:** 2024-09-01

**Authors:** Tom A Yates, Samara Barnes, Martin Dedicoat, Onn Min Kon, Heinke Kunst, Marc Lipman, Kerry A Millington, Andrew J Nunn, Patrick PJ Phillips, Jessica L Potter, S Bertel Squire, on behalf of UK Academics and Professionals to end TB

**Affiliations:** a Institute of Health Informatics, University College London, 222 Euston Road, London NW1 2DA, England.; b UK Academics and Professionals to end TB, United Kingdom of Great Britain and Northern Ireland.; c Department of Infectious Diseases, University Hospitals Birmingham NHS Foundation Trust, Birmingham, England.; d National Heart and Lung Institute, Imperial College London, London, England.; e Blizard Institute, Queen Mary University of London, London, England.; f Faculty of Medical Sciences, University College London, London, England.; g Department of Clinical Sciences, Liverpool School of Tropical Medicine, Liverpool, England.; h Medical Research Council Clinical Trials Unit at UCL, University College London, London, England.; i UCSF Center for Tuberculosis, University of California San Francisco, San Francisco, United States of America.

## Abstract

Most phase III trials in drug-resistant tuberculosis have either been underpowered to quantify differences in microbiological endpoints or have taken up to a decade to complete. Composite primary endpoints, dominated by differences in treatment discontinuation and regimen changes, may mask important differences in treatment failure and relapse. Although new regimens for drug-resistant tuberculosis appear very effective, resistance to new drugs is emerging rapidly. There is a need for shorter, safer and more tolerable regimens, including those active against bedaquiline-resistant tuberculosis. Transitioning from multiple regimen A versus regimen B trials to a single large phase III platform trial would accelerate the acquisition of robust estimates of relative efficacy and safety. Further efficiencies could be achieved by adopting modern adaptive platform designs. Collaboration among trialists, affected community representatives, funders and regulators is essential for developing such a phase III platform trial for drug-resistant tuberculosis treatment regimens.

## Résumé

La majorité des essais de phase III relatifs à la tuberculose pharmacorésistante soit n'étaient pas assez puissants pour quantifier les fluctuations au niveau des critères microbiologiques, soit étaient trop longs, se poursuivant parfois pendant dix ans. Les critères primaires composites, dominés par des différences dans l'interruption du traitement et les changements de schéma, pourraient dissimuler d'importantes variations en termes d'échec thérapeutique et de rechute. Bien que les nouveaux traitements contre la tuberculose pharmacorésistante semblent très efficaces, la résistance aux nouveaux médicaments évolue rapidement. Il est donc nécessaire d'opter pour des traitements plus courts, plus sûrs et mieux tolérés, y compris ceux actifs contre la tuberculose résistant à la bédaquiline. Délaisser la multitude d'essais opposant un schéma de traitement A à un schéma de traitement B pour se diriger vers un unique essai plateforme de phase III de grande envergure permettrait d'obtenir plus vite des estimations solides concernant l'innocuité et l'efficacité relative. En outre, adopter des modèles de plateforme modernes et adaptatifs contribuerait à de meilleures performances. Enfin, la collaboration entre investigateurs, représentants des communautés concernées, bailleurs de fonds et organismes de réglementation est essentielle à l'élaboration de ce type d'essai plateforme de phase III sur les traitements contre la tuberculose pharmacorésistante.

## Resumen

La mayoría de los ensayos en fase III sobre tuberculosis resistente a los fármacos no ha tenido la potencia suficiente para cuantificar diferencias en los criterios de valoración microbiológicos o ha tardado hasta una década en completarse. Los criterios de valoración principales compuestos, dominados por las diferencias en la interrupción del tratamiento y los cambios de régimen, pueden ocultar diferencias importantes en el fracaso del tratamiento y la recaída. Aunque los nuevos regímenes de tratamiento para la tuberculosis resistente a los fármacos parecen muy eficaces, la resistencia a los nuevos fármacos está apareciendo rápidamente. Se necesitan regímenes de tratamiento más cortos, seguros y tolerables, incluidos los activos contra la tuberculosis resistente a la bedaquilina. La transición de múltiples ensayos de régimen A frente a régimen B a un único gran ensayo de plataforma en fase III aceleraría la obtención de estimaciones sólidas de la eficacia y seguridad relativas. Podrían lograrse mayores eficiencias si se adoptaran diseños de plataforma adaptativos modernos. La colaboración entre los autores de los ensayos, los representantes de las comunidades afectadas, los financiadores y los reguladores es esencial para desarrollar un ensayo de plataforma en fase III de este tipo para los regímenes de tratamiento de la tuberculosis resistente a los fármacos.

## ملخص

كانت معظم تجارب المرحلة الثالثة على السل المقاوم للأدوية إما ضعيفة القدرة على اكتشاف الاختلافات في نقاط النهاية الميكروبيولوجية، أو استغرق استكمالها ما يصل إلى عقد من الزمن. أما نقاط النهاية الأولية المركبة، والتي غالبًا ما تخضع للاختلافات في وقف العلاج وتغييرات النظام، فقد تحجب اختلافات مهمة في فشل العلاج والانتكاس. على الرغم من أن النظم الجديدة لعلاج السل المقاوم للأدوية تبدو فعالة للغاية، إلا أن المقاومة للأدوية الجديدة تظهر بسرعة. هناك حاجة إلى نظم علاجية أقصر وأكثر أمانًا وأكثر تحملاً، بما في ذلك تلك النشطة ضد السل المقاوم للبيداكويلين. إن الانتقال من تجارب النظام A المتعددة مقابل تجارب النظام B، إلى تجربة منصة واحدة كبيرة من المرحلة الثالثة، سوف يؤدي إلى تسريع الحصول على تقديرات قوية للفعالية والسلامة النسبية. ويمكن تحقيق المزيد من الكفاءات من خلال انتهاج تصميمات المنصة الحديثة المتكيفة. إن التعاون بين القائمين على التجربة، وممثلي المجتمع المعني، والممولين، والمنظمين يعد ضروريًا لتطوير تجربة منصة تلك المرحلة الثالثة لنظم علاج السل المقاوم للأدوية.

## 摘要

大多数关于耐药结核病的 III 期试验在检测微生物终点差异方面能力不足，或者需要长达十年的时间才能完成。主要复合终点通常以治疗中断和治疗方案改变方面的差异为主，这可能掩盖了治疗失败和复发风险方面的重要差异。尽管新的耐药结核病治疗方案看起来非常有效，但新药很快就会产生耐药性。因此，需要疗程更短、安全性更高、耐受性更好的治疗方案，包括那些对贝达喹啉耐药结核病有效的治疗方案。从方案 A 与方案 B 的多项对比试验过渡到单个大型 III 期平台试验将加速获得对相对疗效和安全性的稳健估计量。可以通过采用现代自适应平台设计，进一步提高效率。试验人员、受影响的社区代表、资助者和监管机构之间的合作对于这种开发耐药结核病治疗方案的 III 期平台试验至关重要**。**

## Резюме

В большинстве случаев статистическая мощность испытаний фазы III при лекарственно-устойчивом туберкулезе не позволяет выявить различия в микробиологических конечных точках, а проведение достаточно мощных исследований занимает до десяти лет. Составные первичные конечные точки, в которых часто доминируют различия в прекращении лечения и смене схемы, могут маскировать важные различия в показателях безуспешного лечения и рецидивов. Несмотря на эффективность новых схем лечения лекарственно-устойчивого туберкулеза, устойчивость к новым препаратам развивается быстро. Необходимо разработать более короткие, безопасные и переносимые схемы лечения, в том числе активные против туберкулеза, устойчивого к бедахилину. Переход от нескольких испытаний схемы лечения A по сравнению со схемой B к одному большому платформенному испытанию фазы III ускорит получение надежных оценок относительной эффективности и безопасности. Дополнительной эффективности можно добиться за счет применения современных адаптивных конструкций платформ. Сотрудничество между исследователями, представителями затронутых сообществ, финансирующими и регулирующими органами имеет важное значение для разработки таких платформенных испытаний схем лечения лекарственно-устойчивого туберкулеза фазы ІІІ.

## Introduction

Rifampicin and isoniazid are key drugs in treatment regimens for drug-susceptible tuberculosis. Rifampicin-resistant tuberculosis and multidrug-resistant tuberculosis (tuberculosis which is resistant to both isoniazid and rifampicin) are managed similarly, and rifampicin resistance is usually accompanied by isoniazid resistance.^1^^,^^2^

In 2022, an estimated 410 000 people worldwide developed rifampicin-resistant tuberculosis yet only 43% started an appropriate treatment regimen.^3^ Among those treated, treatment success for rifampicin-resistant tuberculosis (63%) was lower than that for drug-susceptible tuberculosis (88%).^3^ In 2022, rifampicin-resistant tuberculosis caused an estimated 160 000 deaths.^3^

The December 2022 update to the World Health Organization (WHO) consolidated guidelines on tuberculosis endorses several treatment regimens for rifampicin-resistant tuberculosis.^1^ These include 6- and 9-month all-oral bedaquiline-based regimens, plus longer bespoke regimens for patients not eligible for the shorter regimens, such as people with drug allergies.^1^ Practice varies globally, with many treatment programmes unable to access component drugs included in the new shorter regimens.

While these new regimens mean treatment duration has reduced over recent years (from 18–24 months to 6–9 months), they are not without problems. They include drugs with substantial adverse effects.^4^ They may also have too low a genetic barrier to developing acquired resistance.

Bedaquiline is a key component of modern rifampicin-resistant tuberculosis treatment. People taking bedaquiline-based regimens differ in several ways from people taking rifampicin-based regimens, primarily because they usually have rifampicin-resistant tuberculosis. However, under programmatic conditions, people taking bedaquiline-based regimens appear to acquire drug resistance more than 10 times faster than those on rifampicin-based regimens.^5^^–^^8^ This higher rate of acquired drug resistance is a particular problem given the lack of rapid, near-patient drug susceptibility testing for bedaquiline and other newer drugs.^9^

Future studies need to address critical unanswered questions relating to the treatment of drug-resistant tuberculosis. These include: (i) the relative efficacy, tolerability and safety of the various shorter regimens that were evaluated in recent randomized controlled trials;^10^^–^^14^ (ii) optimum treatment duration (which is likely to vary by patient characteristics);^15^ and (iii) how best to manage people with bedaquiline-resistant tuberculosis.

In this paper, we argue that robust estimates of the relative efficacy, tolerability and safety of rifampicin-resistant tuberculosis treatment regimens are best obtained from randomized controlled trials; that primary efficacy endpoints in these trials should centre on treatment failure and relapse; that estimating the relative frequency of these outcomes with sufficient precision will require better-powered trials and that, while greater capacity to undertake treatment trials in rifampicin-resistant tuberculosis is needed, substantial improvements in study power might be achieved by transitioning from multiple smaller phase III trials to a single platform trial.

While we concentrate on rifampicin-resistant tuberculosis, similar arguments might be made for other forms of tuberculosis, including drug-susceptible tuberculosis and tuberculous meningitis.

## Current approaches, and their limitations

Treatment regimens for drug-susceptible tuberculosis were developed in a succession of highly successful randomized controlled trials^16^ but, until recently, treatment recommendations in rifampicin-resistant tuberculosis have been based on cohort studies^17^^–^^19^ and expert opinion. In tuberculosis treatment, as in other areas of medicine, findings from cohort studies and expert opinion have subsequently been refuted by evidence from randomized controlled trials. For example, a meta-analysis of observational data suggesting use of aminoglycosides in multidrug-resistant tuberculosis regimens was associated with worse outcomes^18^ resulted in guideline changes. This conclusion is not consistent with the results of a subsequent randomized controlled trial where, in an exploratory analysis, a 28-week bedaquiline-based regimen that included 8 weeks of an aminoglycoside appeared superior to a 40-week bedaquiline-based all oral regimen.^13^ People with rifampicin-resistant tuberculosis, clinicians and treatment programmes might choose to avoid aminoglycosides due to adverse effects, poor tolerability or programmatic considerations, but reliable estimates of relative efficacy are needed when making difficult risk–benefit decisions. Similarly, in drug-susceptible tuberculosis, guidelines based on expert opinion included rifampicin-sparing treatment regimens that were subsequently demonstrated to be inferior in a randomized controlled trial.^20^

In recent years, we have seen both the first new drugs for tuberculosis in decades and publication of the first phase III randomized controlled trials of rifampicin-resistant tuberculosis treatment regimens.^10^^–^^14^^,^^21^ These are hugely welcome developments that have had a direct impact upon the lives of people with rifampicin-resistant tuberculosis. Most of these trials have involved comparing a small number of investigational regimens against a single standard of care arm, with the standard of care often changing mid-trial in response to guideline changes.^22^^,^^23^

Most of these trials have not been powered to estimate between-regimen differences in key outcomes, such as treatment failure and relapse (Table 1). The STREAM trials were larger and so better powered than other drug-resistant tuberculosis treatment trials.^13^^,^^21^ However, they each took around a decade from conception to completion of follow-up. The long duration meant that some countries had adopted newer regimens by the time trial results were reported. Increasing the power of randomized controlled trials of rifampicin-resistant tuberculosis treatment regimens could allow modest differences in key efficacy outcomes to be quantified and, also, allow estimation of between-arm differences in the frequency of important but less common safety outcomes.

**Table 1:** Unfavourable events included in the primary outcome in published rifampicin-resistant tuberculosis treatment trials

Trial, regimen, duration	Denominator (modified intention to treat)	Unfavourable events included in the primary outcome, no. (%)			
		Microbiological failure	Relapse	Death	Other
**STREAM (stage 1)** ^21^					
Intervention (including injectables)					
9–11 months	245	19 (7.8)	7 (2.9)	9 (3.7)^a^	17 (6.9)
Standard of care	124	7 (5.6)	0 (0.0)	5 (4.0)^a^	13 (10.5)
**NExT** ^14^					
Intervention arm (all oral)					
6–9 months	49	3 (6.1)	1 (2.0)^b^	4 (8.2)	16 (32.7)
Standard of care	44	5 (11.4)	1 (2.3)^b^	4 (9.1)	24 (54.5)
**ZeNix**^10^ **(all arms bedaquiline + pretomanid**, **6 months)**					
Linezolid 1200mg					
26 weeks	44	0 (0.0)	0 (0.0)	0 (0.0)	3 (6.8)
9 weeks	45	0 (0.0)	2 (4.4)	0 (0.0)^a^	3 (6.7)
Linezolid 600mg					
26 weeks	45	0 (0.0)	1 (2.2)	0 (0.0)	3 (6.7)
9 weeks	44	1 (2.3)	1 (2.3)	0 (0.0)	5 (11.4)
**TB PRACTECAL**^11^ **(72-week results)**					
Bedaquiline + pretomanid + linezolid + moxifloxacin					
6 months	138	0 (0.0)	1 (0.7)^c^	0 (0.0)	15 (10.9)
Bedaquiline + pretomanid + linezolid + clofazimine					
6 months	115	1 (0.9)	5 (4.3)^c^	1 (0.9)	9 (7.8)
Bedaquiline + pretomanid + linezolid					
6 months	111	0 (0.0)	3 (2.7)^c^	1 (0.9)	11 (9.9)
Standard of care	137	0 (0.0)	0 (0.0)	5 (3.6)	51 (37.2)
**STREAM (stage 2)** ^13^					
Intervention arm (all oral)					
9 months	196	6 (3.1)	2 (1.0)	3 (1.5)^a^	23 (11.7)
Intervention arm (including injectables)					
6 months	134	2 (1.5)	1 (0.7)	2 (1.5)	7 (5.2)
Standard of care^d^	187	19 (10.2)	1 (0.5)	1 (0.5)^a^	33 (17.6)
**endTB** ^12^					
Bedaquiline + linezolid + moxifloxacin + pyrazinamide					
9 months	118	1 (0.8)	0 (0.0)	2 (1.7)	10 (8.5)
Bedaquiline + clofazimine + linezolid + levofloxacin + pyrazinamide					
9 months	115	2 (1.7)	0 (0.0)	1 (0.9)	8 (7.0)
Bedaquiline + delamanid + linezolid + levofloxacin + pyrazinamide					
9 months	122	1 (0.8)	0 (0.0)	3 (2.5)	14 (11.5)
Delamanid + clofazimine + linezolid + levofloxacin + pyrazinamide					
9 months	118	10 (8.5)	1 (0.8)^c^	3 (2.5)	11 (9.3)
Delamanid + clofazimine + moxifloxacin + pyrazinamide					
9 months	104	4 (3.8)	2 (1.9)^c^	2 (1.9)	7 (6.7)
Standard of care	119	1 (0.8)	0 (0.0)	2 (1.7)	20 (16.8)

^a^ There were additional deaths among participants with microbiological failure or relapse.

^b^ In this trial, no sequencing was performed to distinguish relapse from reinfection.

^c^ Sequencing to distinguish relapse from reinfection is yet to be reported.

^d^ Not all these controls were used in estimating the relative efficacy of the 6-month regimen.

In recent rifampicin-resistant tuberculosis trials, most unfavourable outcomes have been treatment modifications, usually a result of adverse events, or treatment discontinuations (Table 1). While treatment discontinuation is associated with adverse outcomes,^15^ most people with rifampicin-resistant tuberculosis who discontinue treatment a few weeks early will not experience treatment failure or relapse. If early discontinuation does result in treatment failure or relapse, these events will be captured as unfavourable outcomes anyway.

Including treatment modifications and treatment discontinuations in composite primary outcome measures can mask differences in microbiological outcomes. Importantly, where regimens being compared are of different length, including differences in treatment completion within a composite primary outcome will bias results in favour of the shorter regimens. This bias arises because people on longer regimens have more opportunity to stop treatment early. Further considerations in the choice of endpoints in drug-resistant tuberculosis randomized controlled trials are discussed in Box 1.

Box 1 **Further considerations in the choice of endpoints in rifampicin-resistant tuberculosis trials**

**Primary endpoints**

Most deaths in recent rifampicin-resistant tuberculosis trials have been unrelated to tuberculosis. In superiority trials, including death in the primary endpoint can, therefore, mask true benefits. Conversely, including deaths unrelated to tuberculosis in the primary endpoint in non-inferiority trials may result in investigators falsely concluding non-inferiority. We would therefore suggest rifampicin-resistant tuberculosis trials use a primary endpoint focused on microbiological endpoints – treatment failure and relapse. In high transmission settings, relapse and reinfection should, ideally, be distinguished using whole genome sequencing. Tuberculosis-related death might be included in the primary outcome, although there is a risk of introducing bias when ascertaining whether deaths are tuberculosis related in an open label trial. Attribution of cause of death is best done by a panel of experts blinded to treatment allocation.

**Secondary endpoints**

All-cause mortality, treatment modifications, treatment completion and severe adverse events can be included as secondary or safety endpoints. However, depending on the estimand of interest, changes to allocated treatment regimens should not always be considered unfavourable outcomes in tuberculosis randomized controlled trials.^24^ Another approach is to allocate people to treatment strategies, predefining switches in the event of bacteriological failure or the need to discontinue specific components of a regimen. This design was used in TRUNCATE-TB.^25^ Alternatively, subsequent randomizations can be prespecified, as in an on-going trial in neonatal sepsis (ISRCTN48721236). Events that are reported but not included in the primary outcome can still be included in secondary analyses (secondary estimands), including across-trial comparisons.

**Harmonization**

Harmonization of outcomes is important, as it allows the results of trials to be compared. This requires harmonizing both the events included in composite outcomes and the approach taken to handling post-randomization competing events. Switching from multiple small trials to a single large rifampicin-resistant tuberculosis platform trial would be one means of harmonizing trial outcomes. Alternatively, a common agreed set of outcomes for use in tuberculosis trials could be developed – work towards this is ongoing.^26^^,^^27^

**Economic evaluation**

We advocate embedding economic evaluation within rifampicin-resistant tuberculosis trials, including prospective capture of data on patient quality of life. This information is crucial when deciding which regimens to adopt programmatically.^28^ Research is underway to understand which components of quality-of-life scores matter most to people with drug-resistant tuberculosis.^29^

## The case for a platform trial

Platform trials are randomized controlled trials designed to test several different interventions. Platform trials can be amended, over time, to add new study arms or to abandon interventions that are shown to be futile or harmful.

During the coronavirus disease 2019 (COVID-19) pandemic, people with COVID-19 enrolled rapidly into large platform trials.^30^ Within three months, these trials identified effective therapies and also demonstrated that other widely advocated drugs were ineffective or harmful, thus saving hundreds of thousands of lives.^31^ Similar ambition is needed for rifampicin-resistant tuberculosis.

Trials capacity in rifampicin-resistant tuberculosis remains constrained.^32^ While investment in additional trials capacity is clearly needed, major improvements in efficiency could be achieved though greater collaboration – specifically, transitioning from multiple, small, regimen A versus regimen B randomized controlled trials to a single, large adaptive platform trial powered to explore differences in treatment failure and relapse.

The expected gains in efficiency would be both statistical and operational. First, use of common comparator arm(s) would negate the need to enrol separate sets of patients to control regimens for each new investigational regimen tested. Second, trial sites would not lie fallow between studies and could recruit continuously. Often, when trials end, highly trained staff at study sites move on to other projects or jobs resulting in loss of critical trial-specific expertise. The continuity in funding that a platform trial affords would help retain them. Third, new investigational treatment regimens could be added to the platform trial via amendments to existing ethical and institutional approvals, avoiding the need for separate time-consuming applications. Finally, efficiencies of scale can likely be achieved with respect to laboratory work, monitoring and other aspects of trial management.

Additional advantages of a single, large, rifampicin-resistant tuberculosis platform trial include immediate harmonization of trial outcomes and, potentially, allowing direct comparisons to be made between the novel regimens being tested.

## Designing a drug-resistant tuberculosis platform trial

The STREAM trials were two of the largest randomized controlled trials of treatment regimens for rifampicin-resistant tuberculosis.^13^^,^^21^ We argue here that primary efficacy outcomes in such trials should focus on microbiological endpoints. The probability of treatment failure or relapse by week 76, with either of the STREAM stage 2 intervention regimens, was approximately 2%.^13^^,^^33^ As it will be hard to improve on this efficacy, we expect a future rifampicin-resistant tuberculosis platform trial to use a non-inferiority design with respect to the primary efficacy endpoint. However, we would expect that the platform trial is designed to also assess whether new regimens are superior with respect to safety and tolerability endpoints.

Developments in adaptive trial design offer major opportunities to improve the efficiency of platform trials.^12^^,^^34^^–^^38^ These include the possibility of seamless phase II/III studies with an efficient and statistically-principled approach to selecting, from a range of possible interventions, those most likely to succeed.^11^^,^^38^

Several of these new trial designs could be considered for a future rifampicin-resistant tuberculosis platform trial. In our view, the Personalized Randomized Controlled Trial (PRACTical)^37^ could be a good choice. With this design, a set of possible regimens are defined for each participant, considering factors such as baseline drug resistance, contraindications to specific drugs, patient preferences, and whether the local tuberculosis programme can deliver a particular regimen. The participant is then randomized to one of this set of potential regimens. In analysing the results, indirect comparisons are achieved using an approach similar to network meta-analysis. The design maintains most of the advantages of a standard randomized controlled trial with respect to robust estimation of the relative efficacy and safety of treatment regimens. Importantly, the PRACTical design does not require a single standard of care arm to be defined. This approach is a major advantage for rifampicin-resistant tuberculosis trials, given the standard of care has changed frequently in recent years, often requiring changes to the control arm mid-study.^22^^,^^23^ Furthermore, people can be enrolled into a PRACTical trial if able to take any two of the study regimens, which reduces the number of people ineligible to participate.

## Potential disadvantages

While platform trials have the potential to save lives, as seen during the COVID-19 pandemic,^31^ there are potential downsides. In a review of the implementation of platform trials in low- and middle-income countries, the authors describe tensions in balancing the need for a universal study protocol and adapting interventions to best meet local needs.^39^ The authors also highlight the risk that, without concurrent capacity-strengthening, limited research resources in low- and middle-income countries might become dominated by platform trials.^39^ As in other areas of research, transnational platform trials involving research institutions in both high-income countries and low- and middle-income countries are often unequal.^39^ The shift from multiple small, sometimes locally led,^14^ randomized controlled trials, to a smaller number of platform trials clearly entails some loss of pluralism in methods.^39^ If, as is often the case, leadership and decision-making is dominated by researchers from high-income countries, the approach we advocate could exacerbate inequalities and risk producing research that is less relevant in the low- and middle-income country settings where most people with drug-resistant tuberculosis live.^39^ Finally, while a single platform trial should improve efficiency, adaptive platform trials are operationally challenging.^40^^–^^42^

## Next steps

Platform trials were becoming more frequent before the COVID-19 pandemic,^43^ and their wide and successful use during the pandemic^30^ will likely result in the approach being increasingly adopted across a range of disease areas. Something similar to a platform trial has already been tried in rifampicin-resistant tuberculosis. Between 2017 and 2021, the endTB trial recruited 754 people with rifampicin-resistant tuberculosis and randomized them to one of five investigational regimens or standard of care.^12^ Separately testing each of these five novel regimens against standard of care would have required enrolling many hundreds of additional participants. Further efficiencies could be achieved by continuing to enrol participants, by adding new investigational treatment arms, and through collaboration with investigators contemplating inefficient regimen A versus regimen B trials. It may be possible to factorially randomize to both drug-resistant tuberculosis treatment regimens and host-directed therapies, thereby improving the efficiency of the trials.

Achieving a rifampicin-resistant tuberculosis platform trial requires collaboration among researchers. This collaboration includes a commitment to rethinking planned regimen A versus regimen B trials, and observational studies that would consume the limited resources^32^ available for rifampicin-resistant tuberculosis therapeutics research. Cooperation can be advocated by affected community representatives and incentivized by funders issuing specific calls for a consortium to run a rifampicin-resistant tuberculosis platform trial, including a commitment to funding the platform over the long term. An approach to ensuring these compromises do not limit career progression, which has been used in other disease areas, is to assign principal investigator roles within the platform trial for each regimen or comparison. For the reasons outlined above, funders should favour consortia led by scientists in countries with a high burden of rifampicin-resistant tuberculosis. Finally, regulators should work with funders and trialists to design the platform in such a way that regimens containing unregistered drugs might be included, with the results of the platform trial generating the data needed to register these new drugs.

Preclinical work being undertaken by the ongoing multinational UNITE4TB project will develop novel tuberculosis treatment regimens, including some that would be active against rifampicin-resistant tuberculosis.^44^ These regimens may eventually need clinical evaluation in definitive phase III randomized controlled trials. Decisions regarding which regimens are included in any rifampicin-resistant tuberculosis platform trial should be made in collaboration with key stakeholders including affected community representatives, clinicians working in high-burden settings and national tuberculosis programmes. The views of Samara Barnes, who has completed a course of treatment for rifampicin-resistant tuberculosis, are presented in Box 2. The acceptability^13^ and cost–effectiveness^28^ of regimens may vary by setting, but access to effective treatment must be globally equitable.

Box 2 **A patient’s personal perspective on rifampicin-resistant tuberculosis platform trials and regimen selection**

Samara Barnes, one of this paper’s co-authors, was treated for rifampicin-resistant tuberculosis in 2016. She is based in the United Kingdom and is Affected Community Co-lead at United Kingdom Academics and Professionals to end TB.

“In 2016, I was diagnosed with rifampicin-resistant pulmonary tuberculosis. This meant that my treatment regimen had to be lengthened by well over a year.

“I had a permanent address, I had transport to be able to visit the hospital and collect prescriptions that could not be dispensed at my local pharmacy, I had a job that allowed me to take time off for appointments, and my life had structure – meaning there were no issues around taking my medication consistently.

“I have, however, worked with people who are homeless; those who are reliant on legal and illegal drugs; alcoholics; people with mental ill health; people for whom English is not their first language; people in bail hotels; people who live in houses of multiple occupancy. The very people who are most likely to get pulmonary tuberculosis are the very people for whom having to take medication for an extended period is most difficult.

“Abiding by your tuberculosis treatment regimen is difficult enough for those with the most secure and stable lives. It leaves you exhausted, so you need somewhere safe to sleep. You lose your appetite, so the food you do manage needs to be nutritious. You may need antihistamines due to allergic reactions to your medication. So just imagine how difficult it is for those who do not have the scaffolding around them to complete their treatment.

“So, I fully support the call for a platform trial. I’m aware that, in choosing regimens to test, there may be a trade-off between shortening regimens/reducing side-effects and efficacy. I am of the opinion that we should be looking for regimens that remain highly efficacious AND are shorter/more tolerable. By reducing the time of treatment, the patient is surely likely to have more success in completing their treatment. When you have drug-resistant tuberculosis, time is of the essence.”

## Conclusion

People with rifampicin-resistant tuberculosis deserve treatment regimens that are robust, effective, short and tolerable, and informed by data from adequately powered randomized controlled trials. These trials should be powered to estimate differences between regimens in the most important outcomes – treatment failure and relapse. A rifampicin-resistant tuberculosis platform trial would represent a step change in trial efficiency. It is now time for the tuberculosis community to come together, so the next rifampicin-resistant tuberculosis trials can report results in this decade, rather than the next.
